# Tea Polyphenol Modulates Mitochondria‐Associated Endoplasmic Reticulum Membrane of Hippocampal Neurons Targeting Grp75 to Ameliorate Memory Impairment in the Aged T2DM Rats

**DOI:** 10.1002/fsn3.70776

**Published:** 2025-08-13

**Authors:** Mengqian Shi, Le Cheng, Chenhui Lv, Wenjuan Feng, Xi Wang, Shuangzhi Chen, Chenyang Li, Lushan Xue, Cheng Zhang, Xuemin Li, Haifeng Zhao

**Affiliations:** ^1^ Nutritional and Food Science Research Institute, Department of Nutrition and Food Hygiene, School of Public Health and Center for Ecological Public Health Security of Yellow River Basin Shanxi Medical University Taiyuan Shanxi People's Republic of China; ^2^ The Second Clinical Medical College of Shanxi Medical University Taiyuan Shanxi People's Republic of China; ^3^ Center for Disease Control and Prevention in Shanxi Province Taiyuan Shanxi People's Republic of China; ^4^ MOE Key Laboratory of Coal Environmental Pathogenicity and Prevention Ministry of Education Taiyuan Shanxi People's Republic of China

**Keywords:** aged, memory, mitochondria‐associated endoplasmic reticulum membrane, T2DM, tea polyphenol

## Abstract

Tea polyphenol (TP), as the most abundant and unique functional substance of tea, has been widely studied for the neuroprotective effects. Previous studies also found that TP improves the memory impairment of the aged T2DM rats, but the underlying molecular mechanism has not been fully clarified. The model of aged T2DM was induced by injecting D‐galactose and STZ intraperitoneally, as well as feeding with a high‐glucose‐fat diet in rats, and D‐galactose and D‐glucose were used in PC12 cells. Memory function, mitochondrial damage, mitochondria‐associated endoplasmic reticulum membrane (MAM), Ca^2+^, and apoptosis were detected to investigate the role of TP and its main functional component EGCG in the association between mitochondria and the endoplasmic reticulum. In addition, to further verify whether EGCG reduces apoptosis by regulating MAM, molecular docking and Grp75‐siRNA were used. TP intervention alleviates memory impairment, improves insulin resistance, downregulates the expressions of the MAM‐related proteins and MAM structure, and reduces apoptosis. Subsequently, EGCG intervention attenuates the interaction of MAM, as p‐IP3R1‐Grp75 and Grp75‐VDAC1, and inhibits the mitochondrial Ca^2+^ level. In the experiments to validate the mechanism, the results showed that EGCG not only directly connects to Grp75 physically, but Grp75‐siRNA combined with EGCG inhibits mitochondrial Ca^2+^ overload and cell apoptosis by regulating MAM. TP modulates MAM of hippocampal neurons targeting Grp75 to ameliorate memory impairment in the aged T2DM rats, which presents fresh molecular perspectives on the neuroprotective role of TP in T2DM‐related memory impairment.

## Graphical abstract

TP and its major active component, EGCG, exerts neuroprotective effects in the aged T2DM state; TP/EGCG targets Grp‐75 to regulate MAM then improves memory impairment.

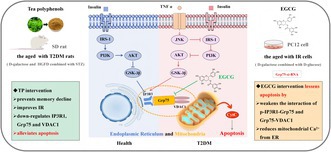

## 1 Introduction

The prevalence of diabetes mellitus (DM) in the elderly has grown to be a serious public health concern as the world's population ages (Lee 2023). The prevalence of DM, especially T2DM, increases with age. The International Diabetes Federation reports that 537 million persons worldwide between the ages of 20 and 79 had diabetes in 2021, with a prevalence rate of 24.0% among those aged 75–79 (Magliano et al. 2021). Patients with DM are prone to develop age‐related comorbidities including cognitive impairment (CI) (Lee 2023). Research has shown that brain dysfunction in DM manifests similarly as it does during CI. A meta‐analysis also showed that 1/15 to 1/10 of the prevalence of CI can be attributed to T2DM (Moheet et al. 2015; Biessels and Reagan 2015). Hence, the glucose uptake/metabolism disorder and insulin resistance (IR) caused by hyperglycemia may be the initial causes of degenerative changes, then induce mitochondrial dysfunction and apoptosis of neurons in T2DM patients' brains (Cassano et al. 2020; Bellia et al. 2022).

As one of the most consumed beverages globally and in China, tea has been found to have potential protective effects against T2DM and CI. A prospective study on chronic diseases in more than 500,000 Chinese showed that the risk of T2DM was reduced by 8% in subjects who drank tea daily compared with those who did not drink tea (Nie et al. 2021). A population survey in Japan and a cross‐sectional study in China about the elderly aged 60 and older found that more green tea consumption lowered the risk of mild CI (Norton et al. 2014). The major component of tea is the tea polyphenols (TP), of which catechins account for approximately 75%–80%. The predominant catechin species is epigallocatechin gallate (EGCG), which makes up 55%–60% of the catechins (Farzaei et al. 2019).

In our previous studies, we have been dedicated to elucidating the ameliorative effects and mechanisms of TP/EGCG on the aged T2DM rat models. We not only discovered that TP/EGCG mitigates damage to the pancreas, kidneys, skeletal muscle, and intestinal barrier in the aged T2DM rat models, but also found that it ameliorates synaptic impairment in the hippocampus by modulating peripheral inflammation driven by gut microbiota, ultimately improving memory deficits (Lv et al. 2024; Wang et al. 2024; Chen et al. 2024). Mechanistically, in recent years, our research on memory impairment has revealed that mitochondrion and the resulting dysfunction in interactions with other organelles within hippocampal neurons may be one of the root causes of impaired neuronal function (Kou et al. 2024; Feng et al. 2024). We have found that the aged T2DM model rats showed glucose uptake/metabolism disorder, and the disorder led to subsequent dysfunction and morphological disorder of mitochondrion in hippocampal neurons (Kou et al. 2024). Another study also found that glucose metabolism disorders interfered with endoplasmic reticulum (ER) function and increased ER stress, thereby affecting mitochondrial autophagy and triggering apoptosis (Feng et al. 2024). Importantly, the above adverse outcomes were reversed with the intervention of TP/EGCG. However, as we further investigated, we found that contact disorder between mitochondria and ER may be the triggering factor for abnormal mitochondrial energy metabolism and apoptosis induced by mitochondrial dysfunction. Therefore, based on the clear understanding of the core role of maintaining mitochondrial function in neuronal activity, we hypothesize that the occurrence of ER stress may alter the normal signaling and contact between the ER and mitochondria, ultimately leading to mitochondrial dysfunction.

The alterations of the contacts between mitochondria and ER are related to many pathological conditions including DM and neurodegenerative diseases (Calvo‐Rodriguez and Bacskai 2021; Barazzuol et al. 2021). Barazzuol et al. (2021) also found that the occurrence of IR leads to increased contact between mitochondria and ER by activating GSK 3β. Structurally, the mitochondria and ER are closely connected intracellular organelles, with the ER membrane forming mitochondria‐associated ER membranes (MAM) on about 5%–20% of the mitochondrial surface. The ER typically encompasses the mitochondria and interacts with them dynamically within a range of < 30 nm, taking physiological functions (Csordás et al. 2018). MAM‐related proteins, IP3R1‐GRP75‐VDAC1 complex, function as a tether to connect the ER and mitochondria, constructing physical interactions. Functionally, a direct and proper Ca^2+^ transport channel from the ER to the mitochondria is provided, which includes inositol 1, 4, 5‐trisphosphate receptor type 1 (IP3R1) in ER, glucose‐regulated protein 75 (Grp75), voltage‐dependent anion channel 1 (VDAC1) in mitochondria, and mitochondrial calcium uniporter (MCU) (Szabadkai et al. 2006). Ca^2+^ serves as an intracellular secondary messenger for transmitting signals in numerous cellular processes of neurons. Basal Ca^2+^ fluctuations in the mitochondria are essential for biological energy production and neuron survival. However, abnormal mitochondrial Ca^2+^ causes insufficient energy production in cells and the release of cytochrome C (Cytc), a proapoptotic factor, which ultimately results in neuron apoptosis (Gomez et al. 2016). Therefore, focusing on MAM of hippocampal neurons not only represents the in‐depth exploration of previous research but may also provide a promising strategy for TP to ameliorate neuronal apoptosis and memory deficits.

Hence, in the current study, based on previously published research, we conduct the experiment to assess whether TP ameliorates memory impairment targeting MAM of hippocampal neurons in the aged T2DM rats, which offers novel insights into the pathogenesis of DM‐related memory impairment and presents fresh molecular perspectives on the neuroprotective role of TP in DM‐related memory impairment.

## 2 Materials and Methods

### 2.1 Animals and Experimental Design

**FIGURE 1:**
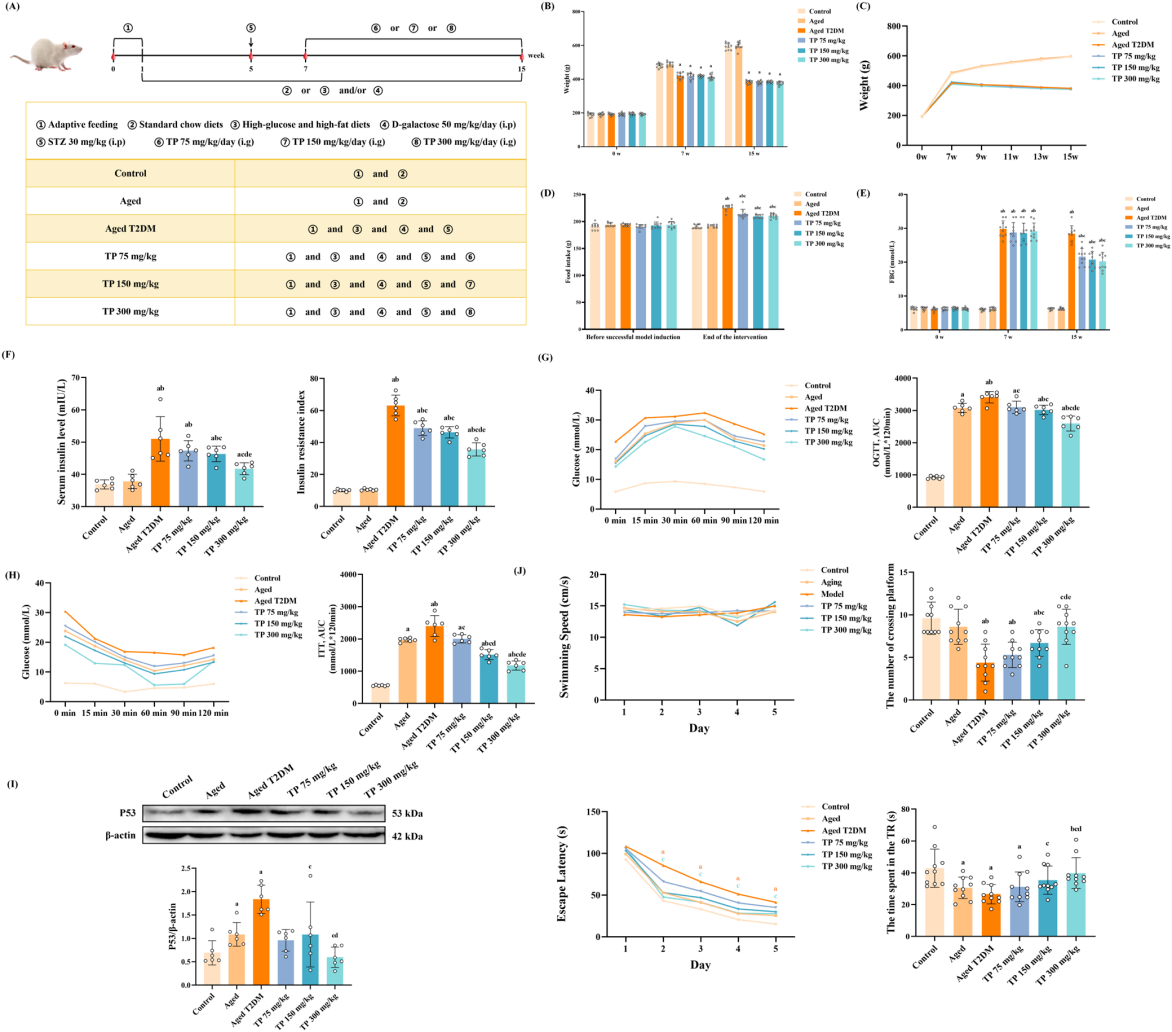
TP ameliorates typical symptoms, downregulates the expression of P53 of the hippocampus, and improves memory impairment of the aged T2DM rat model ($\bar{x}$ ± SD). (A) The schematic diagram of animal experimental design; (B, C) Body weight (*n* = 10); (D) Food intake (*n* = 10); (E) FBG (*n* = 10); (F) Serum insulin level and insulin‐resistant index (*n* = 6); (G) OGTT and the AUC of OGTT (*n* = 6); (H) OGTT and the AUC of OGTT (*n* = 6); (I) The expression of aging‐related protein P53 in the hippocampus (*n* = 6); (J) The average swimming speed, the tendency of escape latency, the time spent in the TR, and the crossing platform times of rats in each group ($\bar{x}$ ± SE, *n* = 10). Data met normality and homogeneity of variance assumptions. One‐way ANOVA with Tukey's post hoc test was used for multiple comparisons. ^a^ *P* < 0.05 versus control group; ^b^ *P* < 0.05 versus aged group; ^c^ *P* < 0.05 versus aged T2DM model group; ^d^ *P* < 0.05 versus TP 75 mg/kg group; ^e^ *P* < 0.05 versus TP 150 mg/kg group.

Male Sprague–Dawley rats (8‐week‐old) were obtained from the China National Institutes for Food and Drug Control (Beijing, China), with the permission number SCXK (Jing) 2022‐0002. The research involving animals follows the ARRIVE guidelines and has been approved by the Institutional Animal Care and Use Committee of Shanxi Cancer Hospital (Issue No. 2022002). Rats were placed in a space with a temperature range of 20°C–26°C and humidity approximately 50% ± 5% during a regular 12 h light–dark cycle. Rats had no restrictions for food and water. After 1 week of acclimatization, 60 rats were randomly assigned to the control group, the aged group, the aged T2DM group (Model), and the intervention group of TP 75 mg/kg (TP‐L), 150 mg/kg (TP‐M), and 300 mg/kg (TP‐H) mg/kg (*n* = 10, respectively). The rats in the model and intervention groups were fed high‐glucose‐high‐fat diets containing 66.5% standard chow, 20% lard, 10% sucrose, 2.5% cholesterol, and 1% sodium cholate. The rats in the control and aged groups were fed the standard chow diet. The protocol for the treatment of rats in each group is depicted in Figure 1A. Rats with FBG levels higher than or equivalent to 16.7 mmol/L on the third, seventh, and fourteenth days following streptozotocin (STZ) intraperitoneal injection were tested for the presence of T2DM. Throughout the trial, rats were weighed weekly and FBG was tested biweekly. At the end of the studies, each group of rats underwent an oral glucose tolerance test (OGTT) and an insulin tolerance test (ITT). OGTT was used to test the glucose tolerance ability after an overnight fast with 1 g/kg glucose orally. ITT was used to test the utilization of glucose after an overnight fast with 0.8 U/kg intraperitoneally. Glucose was detected by the glucometer at 0, 15, 30, 60, 90, and 120 min. The integrated area under the curve for glucose (AUC_glucose_) was used to express the data. HPLC was used to detect TP extracted from green tea (Xi'an lincao Bioengineering, ≥ 98% purity). The primary components of TP were catechin (74%), caffeine (0.8%), ash (3.1%), and others, with EGCG concentration of catechins at 40.2%.

### 2.2 Behavioral Test

The Morris water maze (MWM) test was utilized to assess spatial memory. The maze was set in a silenced space and consisted of a circular black pool (160 × 55 cm) with four quadrants (NE, NW, SW, and SE). The platform (10 cm in diameter) was positioned in the center of NE and submerged 1 cm below the water. The rats were placed in the pool and instructed to find the platform within 120 s and remain there for 10 s. The escape latency (the time that the rats arrived at the platform) was monitored. If the rats were unable to find the platform, the researcher guided the rats for 10 s, and the escape latency was measured at 120 s. All rats were trained for 5 days. On the sixth day, the platform was taken away and the number of times the rats traversed the original platform as well as the staying time in the NE quadrant was documented.

### 2.3 Enzyme‐Linked Immunosorbent Assay (ELISA)

The levels of TNF‐α (Mlbio, China) and insulin (#30589, Mlbio, China) were measured utilizing ELISA. The absorbance at 450 nm was recorded applying the microplate reader (Bio Rad). The Homeostasis Model of Insulin Resistance (HOMA‐IR) index is computed as INS × FBG/22.5.

### 2.4 Transmission Electron Microscopy

1 mm^3^ hippocampus was fixed in electron microscopy fixative solution and placed in 1% osmic acid for 2 h at room temperature in the dark. Then, the hippocampus was dehydrated, infiltrated, and embedded in epoxy resin. For each animal (*n* = 3/group), three nonconsecutive sections were examined and 10 fields per section were randomly captured. The neuronal MAM was observed by transmission electron microscopy (JEM‐1400FLASH) and analyzed the percentage of the length of mitochondria adjacent to ER (the distance between mitochondria and ER is < 30 nm) to the mitochondrial circumference by Image J.

### 2.5 TUNEL

The hippocampus was fixed with a 4% formaldehyde solution for 24 h, then dehydrated, embedded, sectioned, permeabilized, sealed, and stained. Three non‐overlapping fields of hippocampal CA1 area were randomly selected for each prepared section under a 400× magnification microscope. The nuclear pyknosis cells stained brown by TUNEL were determined to be apoptotic cells. For each animal (*n* = 3/group), three nonconsecutive sections were examined, and three fields per section were randomly captured. The number of apoptotic positive cells and total cells in each picture were counted, and the apoptotic index was calculated as the ratio of them.

### 2.6 Cell Culture and Treatment

PC12 cells were cultured in DMEM (Boster, Wuhan, China) added with 10% fetal bovine serum (FBS; Every green, Zhejiang, China) and 1% penicillin/streptomycin (Solarbio, Beijing, China) at 37°C in a 5% CO_2_ incubator. Cells were treated with D‐galactose and D‐glucose in the model group, and the EGCG 5, 10, and 20 μM were added in the intervention group. The procedure for the treatment of cells in each group was depicted in Figure 4A. To inhibit the expression of Grp75, PC12 cells were transfected with short interfering RNA (siRNA, synthesized by Suzhou Ribo Life Science, China), with the riboFECTTM CP Transfection Kit for 48 h, then treated for 24 h. Cells were divided into the control group, the model group, the NC‐siRNA group, the Grp75‐siRNA group, the EGCG 20 μM group, and the Grp75‐siRNA+EGCG 20 μM group. The procedure for the treatment of cells in each group was depicted in Figure 6A.

### 2.7 Senescent β‐Galactosidase (SA‐β‐Gal) Staining

PC12 cells received treatment with SA‐β‐gal working solutions for staining (Servicebio, Wuhan, China) and were cultivated at a constant temperature of 37°C for 2 h. Then, the samples were observed under the light microscope (Olympus, Japan). Results were presented as a proportion of SA‐β‐Gal‐positive cells.

### 2.8 Mitochondrial Ca^2+^ Level

The mitochondrial Ca^2+^ level was evaluated by the Mitochondria Calcium Fluorescence Detection Kit (#GMS10153.1; Genmed Scientifics Inc., USA). The mitochondrial Ca^2+^ concentrations (nmol/L) in the samples were calculated as (RFU of the cell sample well—RFU of the vehicle group) / (RFU of the maximum control well—RFU of the cell sample well)*570 (nmol).

### 2.9 Analysis of the Interactions Between p‐IP3R1, Grp75 and VDAC1

PC12 cells were lysed for 30 min on ice in co‐immunoprecipitation lysis solution including a protease inhibitor cocktail. The lysates were then moved to the microcentrifuge tube and centrifuged (13,000 r/min, 10 min). Anti‐Grp75 antibody was utilized as the bait antibody to capture mitochondria‐ER contact proteins. Co‐IP was carried out with the Co‐IP Kit (ThermoFisher Scientific, USA), and the expressions were evaluated by WB.

### 2.10 Cell Apoptosis

The cells that have been processed were collected and washed with PBS; 250 μL of diluted binding buffer was added (binding buffer: deionized water = 1:9). The 100 μL cell suspension was mixed with 5 μL Annexin V‐FITC and 10 μL PI solution, and then incubated at room temperature for 15 min in the dark. Then 400 μL of 1 × Buffer was added to the reaction tube and detected by flow cytometry. For each condition, three biological replicates (independent cell passages) were tested, with three technical replicates per run.

### 2.11 Western Blot

The protein of hippocampus homogenate was extracted and quantified. Equal total‐protein was separated by 10% SDS‐PAGE, then transferred to PVDF membranes, blocked, and incubated with primary antibodies overnight at 4°C. Further, membranes were washed with PBST for 3 times and incubated with corresponding secondary antibody (anti‐rabbit IgG or anti‐mouse IgG, 1:8000, Boster, China) for 1 h. Washed 3 times in PBST, the membranes were detected by ECL Detection System (Baygene, China) and protein grayscale analysis was measured by Image J software. The primary antibodies included rabbit anti‐p‐JNK (1:1000, #AF3318; Affinity, USA), anti‐JNK (1:1000, #AF6318; Affinity, USA), anti‐p‐IRS‐1 (1:500, #AP0552; Abclonal, China), anti‐IRS‐1 (1:1000, #A0245; Abclonal, China), anti‐p‐GSK 3β (Ser9) (1:1000, #AP0039; Abclonal, China), anti‐p‐GSK3β (Tyr216) (1:1000, #AP0261; Abclonal, China), anti‐IP3R1 (1:1000, #DF3000; Affinity, USA), anti‐p‐IP3R1 (Ser1756) (1:1000, #DF2999; Affinity, USA), anti‐Grp75 (1:500, #AF5464; Affinity, USA), anti‐VDAC1 (1:2000, #GB111939‐100; Servicebio, China), anti‐GSK3β (1:500, #AF5016; Affinity, USA), anti‐MCU (1:1000, #ER1803‐57; Hua'an, China), Cytc (cell signaling technology, USA, 1:1000), p53 (1:1000, #A0263; Abclonal, China) and β‐actin (1:2000, #GB15003; Servicebio, China).

### 2.12 Molecular Docking

In order to clarify whether TP improved apoptosis and memory impairment by targeting MAM, we selected EGCG, the main component of TP, to perform molecular docking with the key connection protein Grp75 of MAM, and explored whether EGCG directly acted on Grp75 structurally. The molecular structure of EGCG is derived from the PubChem compound. The crystal structure of the Grp75 protein was obtained from the Uniprot database. AutoDock Vina software is used for hydrogenation, charge assignment, atom designation, and molecular docking. Data visualization of molecular docking was performed using PyMOL 2.3.2.

### 2.13 Statistical Analysis

Data of the behavioral tests were expressed as mean ± SE. Data of the MWM test was analyzed by ANOVA with repeated measures. For the other data conforming to the normal distribution test, we conducted the homogeneity test of variance, and Tukey's method was used for post hoc analysis. Kruskal–Wallis *H* test and Nemenyi test were used to analyze data following non‐normal distribution. *p* < 0.05 was considered statistically significant.

## 3 Results

### 3.1 TP Ameliorates the Typical Diabetes Symptoms and Downregulated the Expression of Aging‐Related Protein of the Hippocampus in the Aged T2DM Rats

We first observed the effects of TP on the typical symptoms of aged T2DM. The initial body weights showed no significant difference among all the groups (*p* > 0.05). But the weight of rats in the model and TP groups fed with high‐glucose‐high‐fat diets and injected with STZ was significantly reduced at the beginning and the end of the TP intervention compared to the control group (*p* < 0.05) (Figure 1B,C). Besides, at the end of the intervention, the model group had more food intake than the control and the aged groups, and the food intake in the TP groups was decreased in comparison to the model group (*p* < 0.05) (Figure 1D).

Then, the FBG, insulin, HOMA‐IR, OGTT, ITT, and the expressions of P53 in the hippocampus were detected to assess the improvement effect of TP on aging and typical symptoms of T2DM. At 0th w, there was no difference in FBG among the groups, but at 7th w, the model group and TP intervention group showed a significant increase due to high‐glucose‐high‐fat diets and STZ injection, whereas TP intervention suppressed the increase in FBG levels compared to the model group (*p* < 0.05) (Figure 1E). End of the intervention, serum from each group of rats was collected to detect insulin levels and calculate HOMA‐IR. Similar to the FBG results, insulin levels and HOMA‐IR were significantly elevated in the model group rats, but different doses of TP intervention downregulated these indicators, and the TP‐H group had a more significant reduction than the other TP intervention groups (*p* < 0.05) (Figure 1F). Further testing of pancreatic function in each group of rats using OGTT and ITT revealed that the AUC of OGTT and ITT in the model group were decreased when compared with the control group (*p* < 0.05). Compared with the model group, the AUCs of OGTT and ITT in the TP intervention groups were up‐regulated, and the TP‐H group showed significant improvement compared to the other TP intervention groups (*p* < 0.05) (Figure 1G,H).

In order to simulate the physical state of the elderly T2DM patients, based on high‐glucose‐high‐fat diets and STZ injection‐induced T2DM rats, the rats were further injected with D‐gal to accelerate aging. The expressions of aging‐related protein P53 in the D‐gal‐induced‐aged group and model were increased compared to the control group; however, the expression of P53 was higher in the model group than that in the aged group, indicating that T2DM accelerates aging. After TP interventions, the expression of P53 was downregulated (*p* < 0.05) (Figure 1I).

### 3.2 TP Improves Memory Impairment in the Aged T2DM Rats

Subsequently, the MWM test was conducted to investigate whether TP intervention improves memory impairment in the aged T2DM rats, and there was no statistically significant difference in swimming speed among the groups of rats, nor any interaction between training time and groups (*p* > 0.05). Meanwhile, the escape latency showed a shortening trend with the extension of training time, and the trend of each group was consistent. The escape latency prolonged in the model group compared to the control group; TP‐H intervention shortened the escape latency in the next 4 days (*p* < 0.05). For the detection of spatial memory, it was found that the time in the training quadrant (TR) and the number of crossings of the platform in the model group were markedly reduced, whereas TP intervention up‐regulated these two indicators, which provides evidence that TP enhances the spatial memory ability of the aged T2DM rats (Figure 1J).

### 3.3 TP Reduces TNF‐α and Ameliorated the IR of the Hippocampus in the Aged T2DM Rat

**FIGURE 2:**
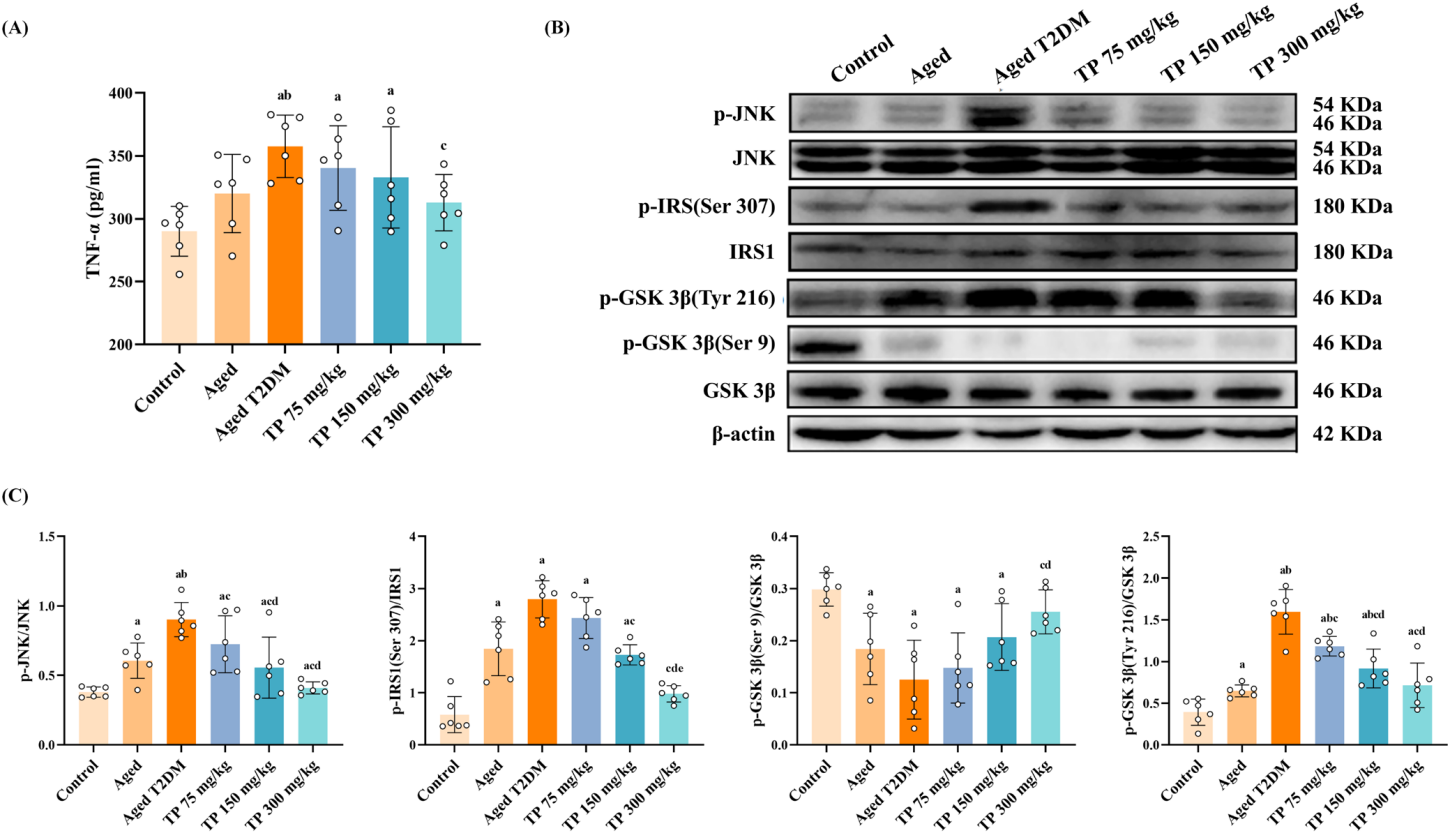
TP reduces TNF‐α and ameliorated the IR of the hippocampus in the aged T2DM rat model ($\bar{x}$ ± SD, *n* = 6). (A) The level of TNF‐α in the hippocampus; (B and C) the expressions of IR‐related proteins in the hippocampus. Data met normality and homogeneity of variance assumptions. One‐way ANOVA with Tukey's post hoc test was used for multiple comparisons. ^a^ *P* < 0.05 versus control group; ^b^ *P* < 0.05 versus aged group; ^c^ *P* < 0.05 versus aged T2DM model group; ^d^ *P* < 0.05 versus TP 75 mg/kg group; ^e^ *P* < 0.05 versus TP 150 mg/kg group.

Higher inflammatory factors and IR induced by sustained hyperglycemia may be the initial causes of CI. Moreover, neuronal IR also leads to mitochondrial dysfunction and apoptosis. Therefore, we first detected expression levels of TNF‐α and IR‐related proteins in the hippocampus. Our results showed that compared with the control group and the aged group, the level of TNF‐α in the hippocampus increased in the model group. However, the level of TNF‐α in the TP‐H group was lower than that of the model group (*p* < 0.05) (Figure 2A). As for the IR signaling pathway, when compared with the control group, the expressions of p‐JNK and p‐IRS1 increased in the aged group and the model group. Although, compared to the model group, the expressions of p‐JNK and p‐IRS1 in the TP groups were downregulated, and the TP‐H group had the strongest effect (*p* < 0.05). The expressions of p‐GSK 3β in Ser‐9 in the aged group and the model group were lower than in the control group. However, the expression of p‐GSK 3β in Tyr‐216 showed an opposite tendency; the expressions of p‐GSK 3β in Tyr‐216 were increased in the aged group and the model group compared to the control group (*p* < 0.05). Although, the expression of p‐GSK 3β in Ser‐9 was up‐regulated in the TP‐H group (*p* < 0.05), and the expression of p‐GSK 3β in Tyr‐216 was decreased in all the intervention groups. The TP‐H group also had the strongest effect (*p* < 0.05) (Figure 2B,C). To sum up, these results indicate that TP reduces inflammation caused by hyperglycemia and improves IR exacerbated by inflammation in the aged T2DM rats.

### 3.4 TP Ameliorates the Structure of MAM in the Hippocampal Neurons of the Aged T2DM Rat

**FIGURE 3:**
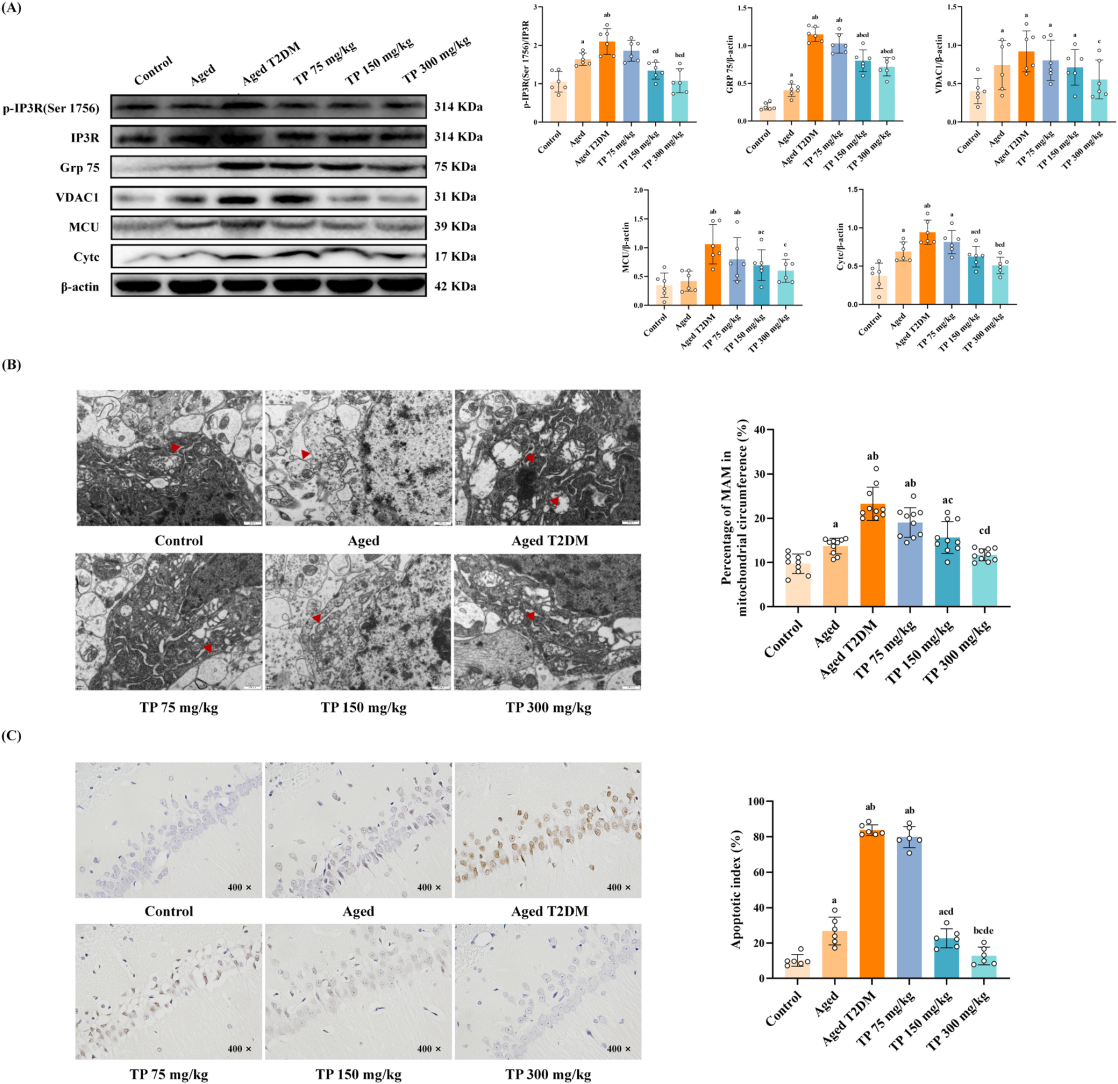
TP ameliorates the structure of MAM and reduced apoptosis of hippocampal neurons in the aged T2DM rat model ($\bar{x}$ ± SD). (A) The expression of MAM‐related proteins and Cytc in the hippocampus (*n* = 6); (B) the ultrastructure of MAM in the hippocampus (scale bar = 500 nm) and quantitative analysis (*n* = 10); (C) the apoptosis of hippocampal neurons reflected by TUNEL (*n* = 3). Data met normality and homogeneity of variance assumptions. One‐way ANOVA with Tukey's post hoc test was used for multiple comparisons. ^a^ *P* < 0.05 versus control group; ^b^ *P* < 0.05 versus aged group; ^c^ *P* < 0.05 versus aged T2DM model group; ^d^ *P* < 0.05 versus TP 75 mg/kg group; ^e^ *P* < 0.05 versus TP 150 mg/kg group.

In previous studies, we have observed the effect of hyperglycemia on ER and mitochondria in the aged T2DM rat, but the direct relationship between them is still unclear. Besides, other studies have also found that IR affected the structure of MAM by activating GSK 3β. The activation of IR and GSK 3β has been observed in the aged T2DM rat; therefore, we further explore the structural changes in direct contact between mitochondria and ER in the hippocampal neurons. We not only detected the expressions of MAM‐related proteins by western blot, but also observed the ultrastructure of neuronal MAM, that is, the distance between mitochondria and ER is < 30 nm using transmission electron microscopy. Our results about the expression of MAM‐related proteins showed that compared with the control group, the expression of p‐IP3R1 in Ser‐1756, Grp75, VDAC1, and MCU increased in the model group (*p* < 0.05). However, the intervention of TP significantly downregulated the expression of these proteins, with the most significant effect observed at 300 mg/kg (*p* < 0.05) (Figure 3A). Our quantitative results based on transmission electron microscopy images revealed that the model group had a higher percentage of mitochondria length adjacent to ER to the mitochondrial circumference than that in the control group (*p* < 0.05). Compared with the model group, the percentage of MAM in mitochondrial perimeter was downregulated in the TP intervention group, especially TP‐M and TP‐H groups (*p* < 0.05) (Figure 3B). Therefore, in our research, it has also been confirmed that the activation of IR and GSK 3β indeed leads to increased contact between mitochondria and ER; however, TP intervention corrects the structure of the MAM of the hippocampal neurons in the aged T2DM rats.

### 3.5 TP Reduces Neuronal Apoptosis of the Hippocampus in the Aged T2DM Rat

The increase of MAM further leads to the influx of massive Ca^2+^ from ER into mitochondria via MAM structure, resulting in the release of CytC and apoptosis. So, we first measured the expression of Cytc, apoptosis‐related protein expression. The results demonstrated that compared with the control group, the expression of Cytc increased in the aged T2DM group (*p* < 0.05). However, compared with the model group, the expression of Cytc was downregulated in the TP intervention groups (*p* < 0.05) (Figure 3A). We then observed apoptosis in the hippocampus of each group of rats by TUNEL staining. Our results showed that TUNEL positive cells, which were brownish yellow or yellowish brown granules in the hippocampus, were fewer and the nuclei of neurons were mostly blue in the control group. Meanwhile, the number of TUNEL positive cells and the apoptosis index (AI) in the model group markedly increased compared to the control group (*p* < 0.05). However, the number of positive cells and AI in the TP intervention groups were downregulated, with the most significant effect observed at the TP‐H group (*p* < 0.05) (Figure 3C). Clearly, all evidence supports that TP reduces apoptosis of the hippocampal neurons in the aged T2DM rats.

### 3.6 The Co‐Exposure of D‐Glucose and D‐Gal in PC12 Cells Induces Aging and IR

**FIGURE 4:**
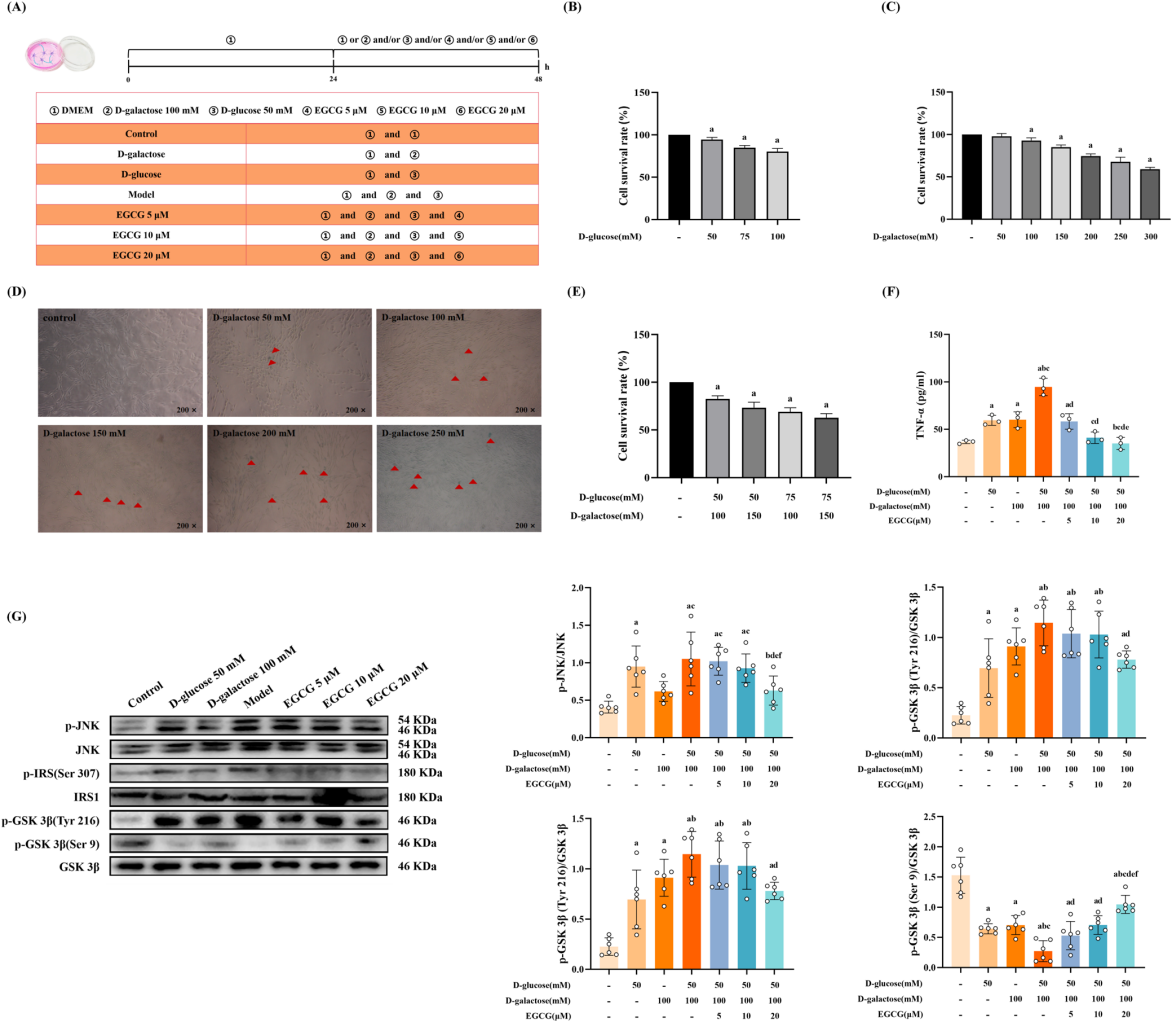
EGCG reduces the level of TNF‐α and ameliorated IR in the model cells co‐exposured to D‐galactose and D‐glucose. (A) The schematic diagram of cell experimental design; (B, C, and E) Determination of the optimal concentration of D‐glucose, D‐galactose, D‐glucose combined with D‐galactose and EGCG in the cells (*n* = 3); (D) Typical SA‐β‐gal staining images of PC12 cells (200 ×, *n* = 3); (F) The level of TNF‐α in the cells; (G) The expressions of IR‐related proteins in the cells. Data met normality and homogeneity of variance assumptions. One‐way ANOVA with Tukey's post hoc test was used for multiple comparisons. ^a^ *P* < 0.05 versus control group; ^b^ *P* < 0.05 versus D‐galactose 50 mM group; ^c^ *P* < 0.05 versus D‐glucose 100 mM group; ^d^ *P* < 0.05 versus model group; ^e^ *P* < 0.05 versus EGCG 5 μM group; ^f^ *P* < 0.05 versus EGCG 10 μM group.

In order to further clarify that TP affects the interaction of MAM and then releases a large amount of Ca^2+^ from ER to mitochondria, resulting in neuronal apoptosis, we conducted the cellular experiments by treating with D‐gal to simulate aged and D‐glucose to simulate hyperglycemia in PC12 cells. Firstly, the appropriate concentration of D‐gal (100, 150, 200, 250 and 300 mM) and D‐glucose (50, 75, 100 mM) was detected by CCK8. To further verify whether D‐gal could induce cellular aging, we performed SA‐β‐gal staining. The results showed that cells were almost free of blue‐stained cells in the control group, whereas blue‐stained cells gradually increased with the increase of D‐gal concentration. Combining the results of the SA‐β‐gal staining and the CCK8, D‐glucose 50 mM and D‐gal 100 mM were used for subsequent experiments (Figure 4B–E).

Then, we explored the improvement effect of different concentrations of EGCG, the main active ingredient of TP, intervention on co‐exposed D‐gal and D‐glucose. The results indicated that compared with the control group, the level of TNF‐α in the model group rose; however, the level of TNF‐α in each intervention group was downregulated, and the level of TNF‐α in the 20 μM EGCG intervention group was lower than that of others (*p* < 0.05) (Figure 4F). IR‐related proteins were detected, and the results revealed that compared with the control group, the expressions of p‐JNK, p‐IRS1 in Ser‐307, and p‐GSK 3β in Tyr‐216 were increased in the model group, and the expression of p‐GSK 3β in Ser‐9 was decreased (*p* < 0.05). After EGCG 20 μM treatment, the expression of p‐JNK, p‐IRS1 in Ser‐307, and p‐GSK 3β in Tyr‐216 was downregulated, and the expression of p‐GSK 3β in Ser‐9 was up‐regulated (*p* < 0.05) (Figure 4G). Given these results, we concluded that in vitro experiments, EGCG intervention reduces the level of TNF‐α and ameliorates IR in D‐galactose and D‐glucose co‐exposed PC12 cells.

### 3.7 EGCG Modulates Mitochondrial‐ER Contacts and Lessens Apoptosis in the Model Cells Co‐Exposed to D‐Gal and D‐Glucose

**FIGURE 5:**
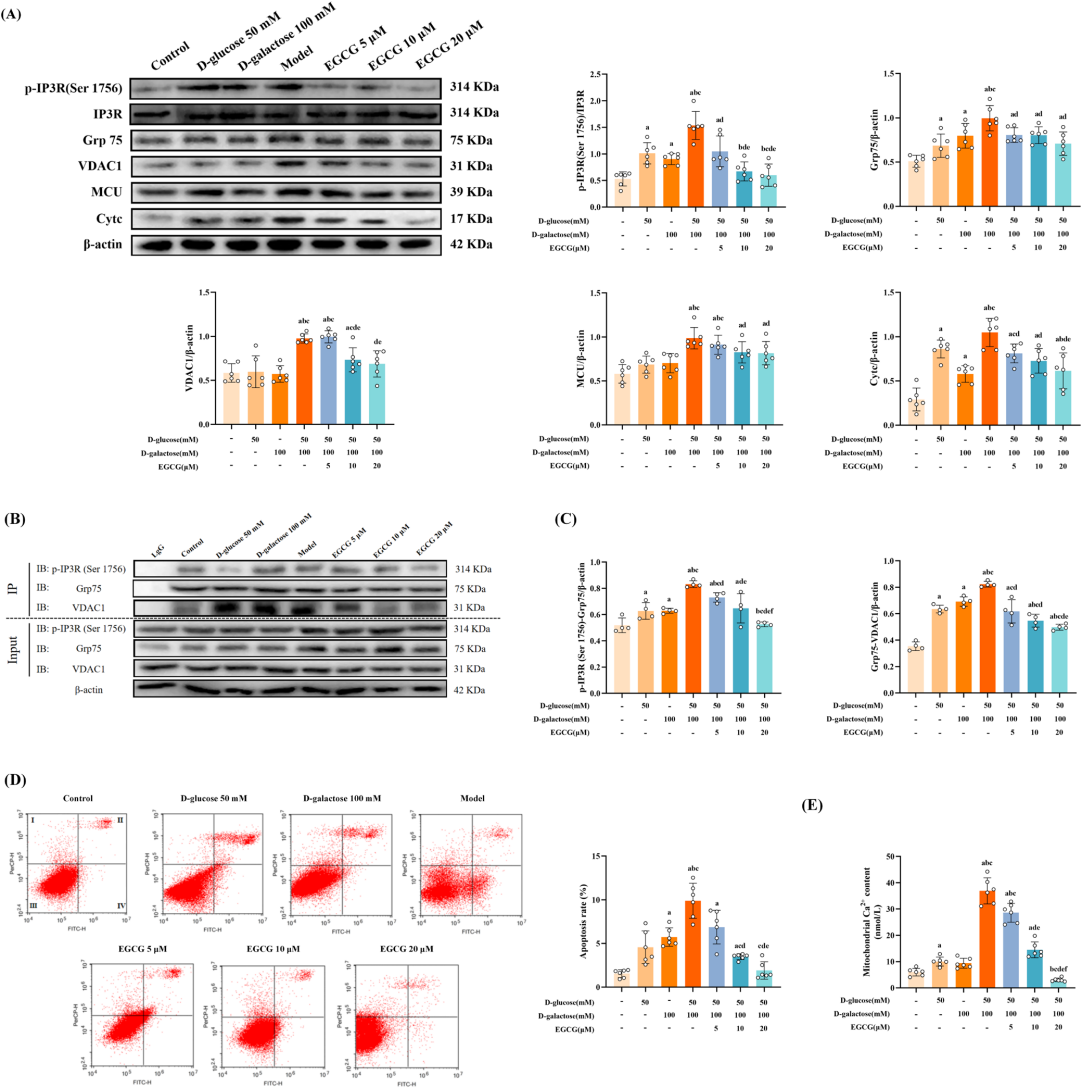
EGCG modulates mitochondrial‐ER contacts and lessens apoptosis in the model cells co‐exposured to D‐galactose and D‐glucose ($\bar{x}$ ± SD). (A) The expression of MAM‐related proteins and Cytc in the cells (*n* = 6); (B, C) The interaction of p‐IP3R1‐Grp75 and the interaction of Grp75‐VDAC1 in the cells (*n* = 4); (D) The apoptosis of PC12 cells reflected by flow cytometry (*n* = 3); (E) Mitochondrial Ca^2+^ levels in the cells (*n* = 3). Data met normality and homogeneity of variance assumptions. One‐way ANOVA with Tukey's post hoc test was used for multiple comparisons. ^a^ *P* < 0.05 versus control group; ^b^ *P* < 0.05 versus D‐galactose 50 mM group; ^c^ *P* < 0.05 versus D‐glucose 100 mM group; ^d^ *P* < 0.05 versus model group; ^e^ *P* < 0.05 versus EGCG 5 μM group; ^f^ *P* < 0.05 versus EGCG 10 μM group.

Firstly, we observed mitochondrial‐ER contacts from both structural and functional perspectives. In terms of structure, we tested the expression of MAM‐related proteins, and the results indicated that compared with the control group, the expressions of IP3R1, Grp75, VDAC1, and MCU increased in the model group (*p* < 0.05). The expressions of the above proteins were downregulated after the EGCG intervention, and EGCG 20 μM showed a more significant effect (*p* < 0.05) (Figure 5A). As for function, we not only observed the interaction of p‐IP3R1‐Grp75 and the interaction of Grp75‐VDAC1, but also detected Ca^2+^ levels in the mitochondria of model cells. The results of the former showed that compared with the control group, the interaction of p‐IP3R1‐Grp75 was stronger in the model group (*p* < 0.05). Compared with the model group, the interaction of p‐IP3R1‐Grp75 and the interaction of Grp75‐VDAC1 were decreased in the EGCG intervention groups, and the EGCG 20 μM group was more effective than the other EGCG intervention groups (*p* < 0.05) (Figure 5B,C). Furthermore, findings of Ca^2+^ levels in the mitochondria indicated that the mitochondrial Ca^2+^ level in the model group increased compared with that in the control group (*p* < 0.05). Compared with the model group and other intervention groups, the mitochondrial Ca^2+^ level in the EGCG 10, 20 μM group was significantly downregulated (Figure 5E).

Then, we assessed the expression of apoptosis‐related protein Cytc. When compared with the control group, the expression was increased in the model group (*p* < 0.05). However, EGCG intervention reduced the expression of Cytc (*p* < 0.05) (Figure 5A). The excessive release of Cytc is considered an initiating factor for apoptosis. Therefore, we further tested the improvement effect of EGCG intervention at different concentrations on apoptosis. Compared with the control group, the apoptosis rate in the model group was increased (*p* < 0.05). The apoptosis rates of the EGCG 10, 20 μM groups were lower than that of the model group (*p* < 0.05) (Figure 5D). Together, these results indicate that EGCG modulates mitochondrial‐ER contacts by weakening the interaction of p‐IP3R1‐Grp75 and Grp75‐VDAC1 as well as ameliorating the overaccumulation of Ca^2+^ in the mitochondria, ultimately lessening apoptosis in the model cells co‐exposed to D‐gal and D‐glucose.

### 3.8 EGCG Reduces Apoptosis by Regulating Mitochondrial‐ER Contacts in the Model Cells Co‐Exposed to D‐Gal and D‐Glucose and Pretreated With Grp75‐siRNA

**FIGURE 6:**
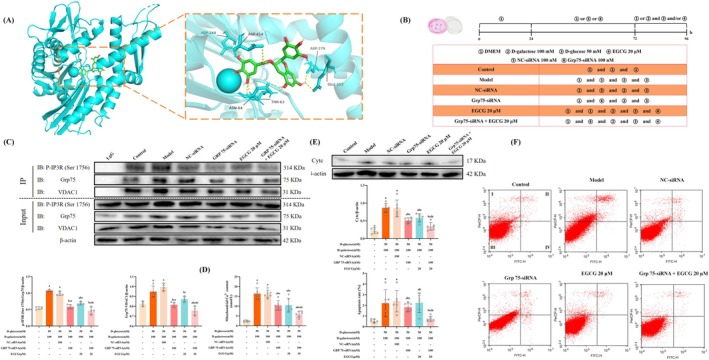
EGCG reduced apoptosis by regulating mitochondrial‐ER contacts in the model cells co‐exposed to D‐galactose and D‐glucose and pretreated with Grp75‐siRNA. (A) Molecular docking predicts the interaction of EGCG with Grp75; (B) the schematic diagram of cell experimental design; (C) the interaction of p‐IP3R1‐Grp75 and the interaction of Grp75‐VDAC1 in the cell (*n* = 4); (D) mitochondrial Ca^2+^ levels in the cells (*n* = 3); (E) the expression of Cytc in the hippocampus (*n* = 6); (F) the apoptosis of PC12 cells reflected by flow cytometry (*n* = 3). Data met normality and homogeneity of variance assumptions. One‐way ANOVA with Tukey's post hoc test was used for multiple comparisons. ^a^ *P* < 0.05 versus control group; ^b^ *P* < 0.05 versus model group; ^c^ *P* < 0.05 versus NC‐siRNA group; ^d^ *P* < 0.05 versus Grp75‐siRNA group; ^e^ *P* < 0.05 versus EGCG 20M group.

To further verify whether TP/EGCG directly targets MAM to inhibit apoptosis, we further validate the effect of TP/EGCG on MAM through molecular docking (Figure 6A) and Grp75‐siRNA (Figure 6B). As the main functional component of TP, EGCG was selected as one of the components for molecular docking. Grp75, as the key connecting protein for MAM, has also been selected as one of the components for molecular docking. The result showed that EGCG had the potential binding capacity to Grp75, and the screening criteria for binding energy < −5.0 kcal/mol were used. The results support that EGCG modulates MAM through Grp75.

Co‐immunoprecipitation results revealed that the interaction of p‐IP3R1‐Grp75 and the interaction of Grp75‐VDAC1 were increased in the model group and the NC‐siRNA compared with the control group (*p* < 0.05). There was no difference in the above indexes between the model group and the NC‐siRNA group (*p* > 0.05). Compared with the model group, the interaction of p‐IP3R1‐Grp75 and the interaction of Grp75‐VDAC1 were weakened in the Grp75‐siRNA, EGCG 20 μM and Grp75‐siRNA + EGCG 20 μM groups (*p* < 0.05). Compared with the Grp75‐siRNA group and the EGCG 20 μM group, the interaction of p‐IP3R1‐Grp75 and the interaction of Grp75‐VDAC1 were weaker in the Grp75‐siRNA + EGCG 20 μM groups (*p* < 0.05) (Figure 6C). Moreover, the mitochondrial Ca^2+^ level in the model group and the NC‐siRNA group increased in contrast with the control group. Compared with the model group, the mitochondrial Ca^2+^ level was downregulated in the Grp75‐siRNA, EGCG 20 μM and Grp75‐siRNA + EGCG 20 μM groups. In addition, compared with the individual treatment group, the combined treatment group showed a more significant reduction (*p* < 0.05) (Figure 6D).

Subsequently, to observe the alteration of cell apoptosis, we not only examined the expression of Cytc, but also conducted flow cytometry. Compared with the control group, the expressions of Cytc increased in the model group and NC‐siRNA group (*p* < 0.05). Compared with the model group, the expressions of Cytc were downregulated in the Grp75‐siRNA, EGCG 20 μM group, and Grp75‐siRNA + EGCG 20 μM group, and the combined treatment group showed a more significant effect (*p* < 0.05) (Figure 6E). For the apoptosis rate, compared with the control group, the apoptosis rates in the model group and the NC‐siRNA group increased (*p* < 0.05). Compared with the model group, the apoptosis rates of the Grp75‐siRNA, EGCG 20 μM and Grp75‐siRNA + EGCG 20 μM groups were reduced. Meanwhile, compared with the individual treatment group, the combined treatment group showed a more significant reduction (*p* < 0.05) (Figure 6F). Taken together, EGCG reduced apoptosis by regulating mitochondrial‐ER contacts in the model cells co‐exposed to D‐gal and D‐glucose and pretreated with Grp75‐siRNA.

## 4 Discussion

As the population ages, the prevalence of T2DM among the elderly has been increasing annually, from 10% in 2000 to 21.1% in 2023 (Jia et al. 2025). Simultaneously, advancements in socioeconomic development and health services have prolonged the lifespan of T2DM patients, leading to complications, including cognitive impairment, which seriously affect health. The guideline for the prevention and treatment of diabetes states that the incidence of dementia in elderly diabetic patients has increased significantly, thus recommending annual early screening for cognitive function in this population, and the expert consensus on cognitive management also suggests that modifiable risk factors for cognitive impairment include exposure and overlap of diabetes and malnutrition, which significantly increase the risk and accelerate the progression of cognitive impairment (Jia et al. 2023). Therefore, it is crucial to prevent and delay the occurrence and development of chronic diseases and their complications among the elderly by addressing these modifiable risk factors as early as possible.

T2DM is characterized by hyperglycemia and IR. More and more studies have shown that T2DM is associated with cognitive dysfunction, especially in the elderly. In a systematic analysis that included several longitudinal studies, having diabetes increases the risk of cognitive decline by 1.2–1.5 times and the risk of dementia by 1.6 times (Cukierman et al. 2005). The neuroprotective benefits of TP have been studied in depth over the past few decades; yet, the potential biological basis of TP to alleviate decline in memory and neuronal apoptosis in aged T2DM states continues to be unanswered. We have confirmed that TP improves mitochondrial dysfunction and mitophagy‐ER stress caused by disordered neuronal glucose metabolism in the hippocampus of aged T2DM rats (Feng et al. 2024; Kou et al. 2024). Interestingly, in the subsequent exploration of the mechanism, we found that the outcomes of mitochondrial energy metabolism disorders and apoptotic processes are actually closely associated with mitochondrial‐ER contact. Therefore, we conducted this study to reveal whether TP targets MAM, thereby mediating mitochondrial dysfunction and neuronal apoptosis, ultimately affecting memory function.

First to all, to simulate the state of the elderly at this stage, we established the aged T2DM model rats by injecting 50 mg/kg D‐gal and 30 mg/kg STZ intraperitoneally and feeding SD male rats with a high‐glucose‐fat diet in vivo experiment. The aged T2DM model cells were established by treating D‐gal and D‐glucose in PC12 cells. D‐gal is considered a recognized agent to induce aging (Azman and Zakaria 2019). STZ could selectively destroy animal islets β‐cells, and intraperitoneal injection of STZ and feeding with a high‐glucose‐fat diet were usually used to establish the T2DM model rat (Zheng et al. 2017). Our results revealed that in vivo, the glucose was higher than or equivalent to 16.7 mmol/L in the aged T2DM rat model compared with the control group, along with increased fasting serum insulin level, HOMA‐IR, and expression of P53 of the hippocampus. In vitro, the SA‐β‐gal staining results showed that in contrast with the control group, the number of blue‐stained cells increased in the D‐gal 50 mM group. The results mentioned above demonstrated that the models were successfully induced.

Epidemiological data shows that DM patients have a higher risk of cognitive dysfunction (Biessels and Reagan 2015). However, the prolonged exposure to hyperglycemia‐mediated inflammation is considered to be the main cause of CI induction. Furthermore, the elevated TNF‐α leads to the suppression of insulin receptor signaling by activating JNK, serine phosphorylation of IRS‐1, and the activation of GSK 3β, eventually affecting neuron apoptosis, which is one of the main pathological features of CI (Biessels and Reagan 2015; Ozcan et al. 2004). Therefore, we first tested the memory function, inflammation, and IR hippocampus in the model rats and found that, in the aged T2DM group, the escape latency prolonged, the time spent in the TR, and the number of crossings of the platform were decreased, suggesting that the aged T2DM rats had memory impairment. The similar results were observed in the aged T2DM rats induced by i.p. injection of STZ in 13 m SD rats and by 25w‐old db/db mice (Fan et al. 2022). In addition, whether in vivo or in vitro experiments, the expression levels of TNF‐α, p‐JNK, p‐IRS1 (Ser 307) and p‐GSK 3β (Tyr 216) increased in the model group, and the expression of p‐GSK 3β (Ser 9) decreased. After TP or EGCG intervention, the above results have been ameliorated. Consistent with our research findings, Yan et al. (2012) pointed out that TP reduced blood glucose levels and enhanced glucose tolerance in animal experiments. EGCG plays an important role in anti‐inflammation and improving IR. EGCG significantly improves IR and cognitive dysfunction by inhibiting TNF‐α in obese C57BL/6J mice. EGCG inhibits high glucose‐induced JNK phosphorylation, enhances insulin signaling, activates IRS1, and reduces GSK 3β activity (Barazzuol et al. 2021; Csordás et al. 2018). TP and EGCG showed the potential effect of ameliorating the high‐fat‐ and high‐fructose‐triggered learning and memory loss (Mi et al. 2017). Furthermore, STZ‐induced DM rats ingested green tea extract (3 mg/L/d) or EGCG (20 or 40 mg/kg/d) via drinking water for 7–8 w, and the memory of the rats was improved (Baluchnejadmojarad and Roghani 2011; Sharifzadeh et al. 2017).

The increase of IR leads to abnormal activation of GSK 3β, resulting in elevated MAM, as mitochondrial‐ER contact. The mitochondria and ER are significant intracellular organelles that actively communicate via structurally and functionally formed contacts. The alterations of the contacts of the mitochondrial and ER are related to a variety of pathological conditions (Nie et al. 2021). IP3R1‐GRP75‐VDAC1 complex controls cell metabolism and Ca^2+^ transport from ER to mitochondria (Calvo‐Rodriguez and Bacskai 2021). As we have previously observed, the impaired neuronal glucose metabolism and apoptosis in the hippocampus of aged T2DM rats are likely caused by the dysregulated complex, which facilitates excessive Ca^2+^ transfer from the ER to mitochondria, which would increase mitochondrial Ca^2+^ influx precipitating the opening of the mitochondrial permeability transition pore (mPTP). In turn, this induces mitochondrial permeability transition, disrupts ATP synthesis, and triggers CytC release, ultimately leading to apoptosis. Therefore, we subsequently performed ultrastructural observations of ER‐mitochondrial contacts in the hippocampus; we found that the percentage of MAM in the mitochondrial perimeter increased in the hippocampus in the aged T2DM group. Both in vivo and in vitro experimental results showed that the expressions of MAM‐related proteins, p‐IP3R1, GRP75, VDAC1, and MCU of the hippocampus in the model group increased. When it comes to function, compared with the control group, the interaction of p‐IP3R1‐Grp75 and the interaction of Grp75‐VDAC1 were stronger in the model group; meanwhile, the mitochondrial Ca^2+^ level in the model group increased, leading to an increase in cell apoptosis. However, after TP and EGCG intervention, the above indexes were improved. Consistent with our research findings, the contacts of mitochondria and ER were significantly increased in presenilin‐mutant cells from AD patients (Area‐Gomez et al. 2012). A study showed that the impairment of mitochondrial calcium efflux capacity in a 3xTg‐AD mouse model led to superoxide accumulation, metabolic dysfunction, and neuronal degeneration (Jadiya et al. 2019). Karthikeyan et al. also unlocked that EGCG attenuated mitochondria‐ER tethering and maintained Ca^2+^ homeostasis compared with the cell group treated with thapsigargin (Karthikeyan et al. 2017).

The molecular chaperone Grp75 is a key protein expressed at the MAM interface and can regulate the Ca^2+^ transfer from ER to mitochondria. Knockdown or pharmacological inhibition of Grp75 reduced the number of interaction sites between IP3R and VDAC1, reducing the MAM and the transfer of Ca^2+^ from the ER to mitochondria, which can prevent cell apoptosis (Honrath et al. 2017). Geng et al. (2025) found that Grp75 deficiency reduced ER‐mitochondria contact, significantly prevented mitochondrial Ca^2+^ overload and mPTP opening, as well as neuronal death in HT22, the mouse immortalized neuronal cells, which further confirmed the central role of Grp75 in MAM structure. Additionally, Chang et al. also revealed that the formation of VDAC1‐GRP75‐IP3R complex and MAM were enhanced in high glucose‐treated SH‐SY5Y, human neuronal cells. However, blocking the interaction of the VDAC1‐GRP75‐IP3R complex alleviated mitochondrial Ca^2+^ overload‐induced mitochondrial dysfunction and apoptosis (Chang et al. 2023). Therefore, we used Grp75‐siRNA transfection to silence the Grp75 protein to verify whether EGCG could regulate mitochondrial Ca^2+^ overload and cell apoptosis caused by MAM abnormality. When siRNA inhibited the expression of Grp75, the results showed that the interaction of p‐IP3R1‐Grp75 and the interaction of Grp75‐VDAC1 decreased, the mitochondrial Ca^2+^ content reduced, and the expression of Cytc and the apoptosis rate declined. However, the Grp75‐siRNA combined with the 20 μM EGCG group had a better effect on improving the above results than that in the Grp75‐siRNA and the 20 μM EGCG group. To sum up, EGCG could regulate mitochondrial Ca^2+^ overload and cell apoptosis caused by MAM abnormality.

Although this study ultimately demonstrated that TP/EGCG ameliorates memory impairment in aged T2DM rats by suppressing neuronal apoptosis targeting Grp75‐mediated modulation of MAM structure, several limitations should be acknowledged. First, we did not systematically investigate the temporal dynamics of MAM structural changes following TP/EGCG intervention, leaving the acute versus chronic nature of this regulation unclear. Future studies should incorporate multiple time‐point assessments to delineate the chronological pattern of MAM remodeling. Second, the study focused exclusively on TP/EGCG without comparative analysis with other known polyphenols which reported could modulate MAM, potentially overlooking compound‐specific mechanisms. In addition, the clinical translational relevance of Grp75 inhibition by EGCG requires additional validation, including its inhibitory efficacy in humans, dose‐dependent safety profile, and blood–brain barrier permeability. The inherently low bioavailability of EGCG may constrain its therapeutic potential, necessitating delivery system optimization. Moreover, whereas Grp75 was our primary focus, which accurately controls the Ca^2+^ flow from ER to mitochondria, we did not examine whether TP/EGCG influences other critical MAM‐associated proteins (e.g., MFN2, PACS‐2, VDAC1), whose potential synergistic or antagonistic interactions might impact the overall antiapoptotic outcome. Additionally, although negative controls were implemented to partially account for potential off‐target effects of Grp75‐siRNA, comprehensive transcriptome sequencing would provide more rigorous evaluation. Addressing these aspects will enable more thorough elucidation of TP/EGCG's neuroprotective mechanisms and facilitate clinical translation.

In conclusion, our research revealed that TP and its major active component, EGCG, exert neuroprotective effects in the aged T2DM state by improving IR, targeting Grp75 to modulate excessive mitochondrial‐ER contacts, and reducing cell apoptosis. These findings provide mechanistic insights into the role of TP in preventive and therapeutic intervention for cognitive decline in elderly diabetic populations and offer evidence for dietary strategies to mitigate diabetes‐related complications and neurodegeneration.

## Author Contributions

**Mengqian Shi:** writing – original draft (equal). **Le Cheng:** writing – original draft (equal), writing – review and editing (equal). **Chenhui Lv:** data curation (equal), formal analysis (equal). **Wenjuan Feng:** data curation (equal), formal analysis (equal). **Xi Wang:** methodology (equal), software (equal), validation (equal). **Shuangzhi Chen:** methodology (equal), software (equal), validation (equal). **Chenyang Li:** investigation (equal), project administration (equal). **Lushan Xue:** investigation (equal), project administration (equal). **Cheng Zhang:** investigation (equal), project administration (equal). **Xuemin Li:** conceptualization (equal), validation (equal), visualization (equal). **Haifeng Zhao:** conceptualization (equal), writing – review and editing (equal).

## Conflicts of Interest

The authors declare no conflicts of interest.

## Data Availability

The data and material used to support the findings of this study are available from the corresponding author upon request.
